# Genetically distinct pestiviruses pave the way to improved classical swine fever marker vaccine candidates based on the chimeric pestivirus concept

**DOI:** 10.1080/22221751.2020.1826893

**Published:** 2020-10-03

**Authors:** Alexander Postel, Paul Becher

**Affiliations:** Institute of Virology, Department of Infectious Diseases, University of Veterinary Medicine, Hannover, Germany

**Keywords:** Classical swine fever virus, pestivirus, chimeric virus, attenuation, marker vaccine, DIVA concept, antibody ELISA

## Abstract

Classical swine fever (CSF) is one of the most important viral diseases of pigs. In many countries, the use of vaccines is restricted due to limitations of subunit vaccines with regard to efficacy and onset of protection as well as failure of live vaccines to differentiate infected from vaccinated animals (DIVA principle). Chimeric pestiviruses based on CSF virus (CSFV) and the related bovine viral diarrhea virus (BVDV) have been licensed as live marker vaccines in Europe and Asia, but cross-reactive antibodies can cause problems in DIVA application due to close antigenic relationship. To develop marker vaccine candidates with improved DIVA properties, three chimeric viruses were generated by replacing E^rns^ of CSFV Alfort-Tübingen with homologue proteins of only distantly related pestiviruses. The chimeric viruses “Ra”, “Pro”, and “RaPro” contained E^rns^ sequences of Norway rat and Pronghorn pestiviruses or a combination of both, respectively. In porcine cells, the “Pro” chimera replicated to high titers, while replication of the “Ra” chimera was limited. The “RaPro” chimera showed an intermediate phenotype. All vaccine candidates were attenuated in a vaccination/ challenge trial in pigs, but to different extents. Inoculation induced moderate to high levels of neutralizing antibodies that protected against infection with a genetically heterologous, highly virulent CSFV. Importantly, serum samples of vaccinated animals did not show any cross-reactivity in a CSFV E^rns^ antibody ELISA. In conclusion, the E^rns^ antigen from distantly related pestiviruses can provide a robust serological negative marker for a new generation of improved CSFV marker vaccines based on the chimeric pestivirus concept.

## Introduction

Classical swine fever (CSF) is one of the most important viral diseases in pigs. Causative agent is the classical swine fever virus (CSFV), an RNA virus belonging to the genus *Pestivirus* within the family *Flaviviridae*. CSF is endemic in many pig-rearing countries worldwide and causes tremendous economic losses [1]. These losses can be caused by the virus itself due to high mortality rates in case of highly virulent CSFV strains or by international trade restrictions after notification of an outbreak. Modified live vaccines (MLV) based on attenuated CSFV strains can represent a very powerful tool of CSF control, if vaccination is embedded in an overall concept [2].

In many countries, the use of vaccines, even in emergency situations, is restricted due to limitations of available subunit vaccines with regard to efficacy and onset of protection or due to failure of conventional live vaccines to differentiate infected from vaccinated animals (DIVA principle). The viral glycoprotein E^rns^ is a promising target to establish a discriminating principle based on antibodies. Alternatively, it has been demonstrated in experimental vaccines that deletion of single, highly conserved epitopes in the E2 protein can provide a serological negative marker [3–5]. The concept of a serological negative marker, meaning that vaccinees lack antibodies directed against a certain viral epitope, is mandatory to prove the freedom of disease in a vaccinated animal population.

Different MLV candidates with marker properties have been developed applying modern molecular techniques, but until now, only two have been licensed. In Europe, a chimeric pestivirus, named CP7_E2Alf, was licensed as a marker MLV in 2014. It harbours the E2 coding region of CSFV in the context of the bovine viral diarrhea virus (BVDV) strain CP7 [6]. This chimeric MLV confers good protection and was characterized in detail [7]. However, due to close antigenic relationship of CSFV and BVDV, cross-reactive antibodies may result in false-positive reactions and failure in DIVA discrimination [8–10]. In addition, a chimeric pestivirus, designated Flc-LOM-BE^rns^, was designed based on the live attenuated CSFV vaccine strain LOM. In this CSFV chimera, the complete E^rns^ and signal peptide encoding sequence was replaced by corresponding BVDV sequences [11]. The vaccine was licensed in South Korea in 2017 and can be applied in domestic pigs as well as in wild boar [12]. In China, an inactivated vaccine based on a recombinant baculovirus expressing the CSFV E2 protein has been licensed in 2017 and is used by some pig farms [13]. No information is available whether the E2 subunit vaccine application is accompanied by DIVA testing.

The genetic construction of chimeric pestiviruses has been demonstrated to be one promising strategy for generation of efficient MLV with marker properties. In principle, chimerization of closely related viruses enables to maintain efficient viral replication, but at the same time may be problematic in terms of induction of cross-reactive antibodies. The discovery of pestiviruses being only very distantly related to CSFV provides new options to design improved CSF marker vaccine candidates based on the chimeric pestivirus concept. In the present study, several chimeric CSFV were designed, which differed in their replication properties *in vitro* and *in vivo*. A first animal trial in pigs demonstrated that chimeric vaccine strains expressing only distantly related E^rns^ antigens are an interesting option to provide both an efficient protection against CSF and a robust serological negative marker with a considerably reduced risk to induce cross-reactive antibodies that may interfere with CSF diagnosis.

## Material and methods

### Generation and characterization of marker vaccine candidates

**Figure 1.**
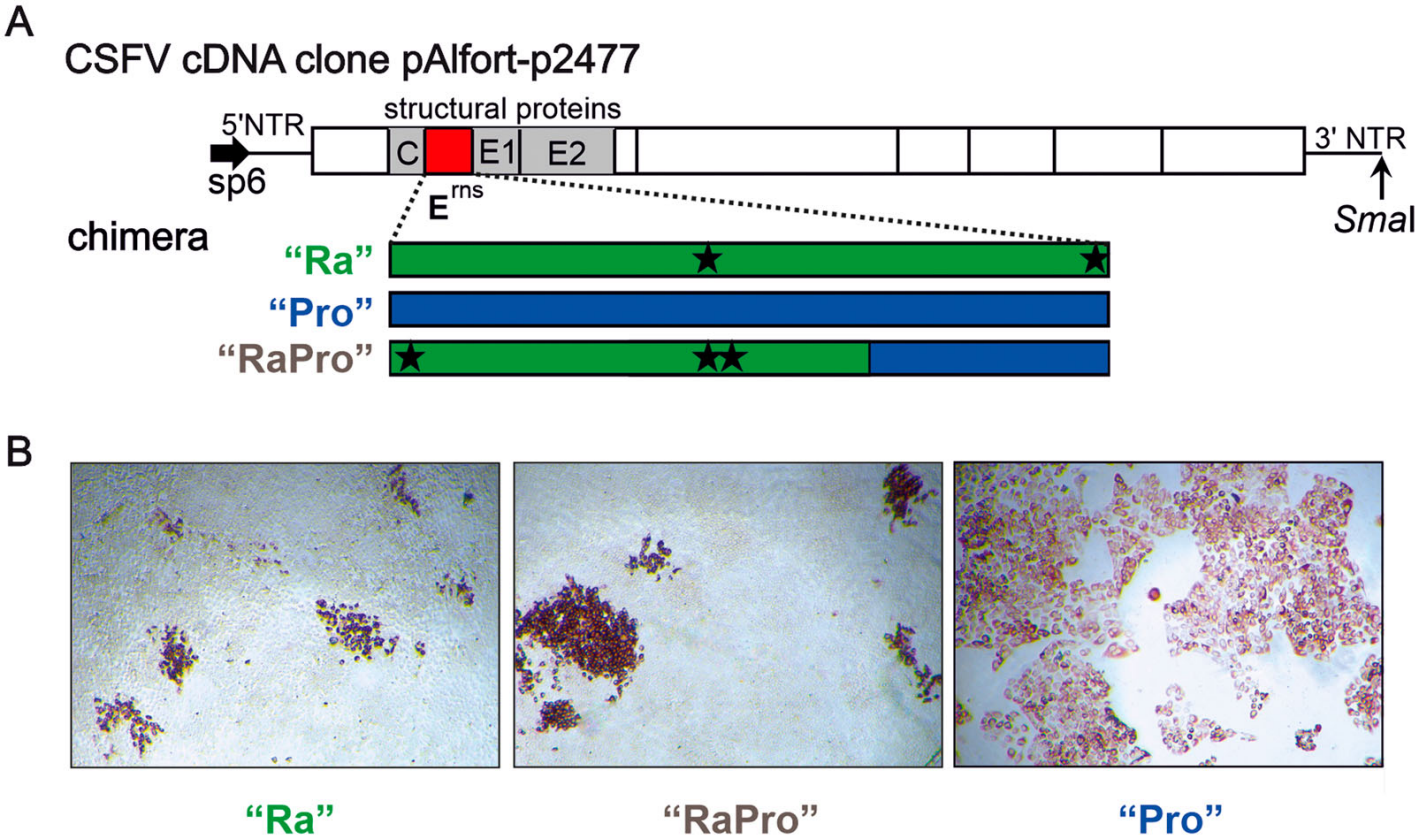
Construction and characterization of chimeric pestiviruses. (A) Genome structure of the chimeric CSFV vaccine candidates. The reverse genetic system allows to generate synthetic genome-like RNAs of CSFV Alfort-p2477 by sp6-driven RNA synthesis after plasmid linearization with *Sma*I. The chimeric derivatives of CSFV designated “Ra”, “Pro” and “RaPro” contain the E^rns^ coding sequences of the Norway rat and Pronghorn pestiviruses or a combination of both, respectively. Adaptive mutations identified in the “Ra” and “RaPro” vaccine stocks are indicated as asterisks. (B) Porcine kidney cell cultures three days after infection with the chimeric pestiviruses “Ra”, “RaPro” and “Pro” and visualization by immunoperoxidase staining with the pestivirus specific mab BVD/C16.

To develop improved CSFV marker vaccine candidates, three chimeric variants were generated by replacing the E^rns^ encoding sequence of a moderately virulent CSFV by the homologous sequences from pestiviruses distantly related to CSFV. A “Ra” chimera (CSFV-2697) containing the E^rns^ sequence of Norway rat pestivirus (NrPV) isolate NYC-D23 (GenBank access. No. NC025677), a “Pro” chimera (CSFV-2677) harbouring the E^rns^ sequence of Pronghorn antelope pestivirus (GenBank NC024018.2), and a “RaPro” chimera combining parts of both E^rns^ sequences (CSFV-2695) were generated (Figure 1). In the “RaPro” construct, the first 492 nucleotides (position 1618–2109) of the chimeric E^rns^ encoding sequence are derived from NrPV followed by 186 nucleotides (position 1660–1845) of the Pronghorn pestivirus sequence. The chimeric pestivirus vaccine candidates were generated on basis of the infectious cDNA clone pAlfort-p447 of CSFV strain Alfort-p447 [14,15]*.* The sequence of the cDNA plasmid used in this study (pAlfort-p2477) is almost identical to the sequence previously published for CSFV Alfort-p447, which contains two additional guanine residues at its 5′ genome end (GenBank LT593760). In addition, pAlfort-p2477 is harbouring three synonymous mutations (4736G > C, 4739C > A, 7616T > C) to generate artificial *BsiW*I and *Kas*I restriction sites, respectively. To produce synthetic viral RNA genomes, pAlfort-p2477 was linearized using a *Sma*I site located at the 3′ end of the CSFV sequence. The *sp6* promoter sequence located directly upstream of the CSFV specific sequence was used for *in vitro* transcription. Synthesized RNA was electroporated into SK6 cells as described previously [16]. Immunofluorescence or immunoperoxidase was performed on heat fixated cells using the broadly reactive pestivirus NS3-specific monoclonal antibody BVD/C16 (diluted 1:25) which was generated and characterized as described previously [17]. For visualization of binding, a Cy3 labelled secondary Goat α-mouse IgG Ab (Dianova, #115-165-003, diluted 1:800) and a polyclonal rabbit anti-mouse horseradish peroxidase conjugate (DAKO, #P0260, dilution 1:200) was used, respectively. Due to impaired growth, “Ra” and “RaPro” chimera were propagated on different cell lines (11 and 13 passages, respectively), including porcine SK6 and PK15 cells as well as ovine SFTR cells, to allow adaptive mutations in the chimeric genomes. Virus stocks used in the animal experiment were genetically characterized by High Throughput Sequencing (HTS) on an Illumina platform as described previously [18]. The vaccine virus candidates and the CSFV strains Alfort-p2477 and Koslov were titrated as described previously [19].

### Animal experiment

The animal experiment consisted of five study groups comprising twenty-three 10 weeks old hybrid pigs obtained from a commercial farm. Three study groups of five pigs were inoculated with the vaccine candidates “Ra”, “Pro”, and “RaPro”, respectively. One additional group of five pigs inoculated with Alfort-p2477 served as attenuation control to be able to monitor the extent of attenuation of the chimeric vaccine candidates depending on the inserted E^rns^ sequence. The attenuation control group was not challenged. Challenge infection of the other groups was performed with the genetically hetero­logous CSFV strain Koslov (genotype 1.1) which has been often used in vaccination/challenge trials due to its extraordinary high virulence. The unvaccinated control group (challenge control) consisted of three pigs to demonstrate that the virus stock of CSFV strain Koslov used for challenge infection is able to induce severe disease under the experimental conditions. As CSFV Koslov usually leads to death of all inoculated pigs within less than one week, the experiment was stopped at 12 and 13 days post challenge (dpc). Post-mortem investigation included the visual control of all organs for signs typically seen as consequence of CSF and sampling of tissues, including kidney, spleen, mandibular lymph nodes and parotic gland.

### Clinical investigations, sampling and determination of blood cell parameters

**Figure 2.**
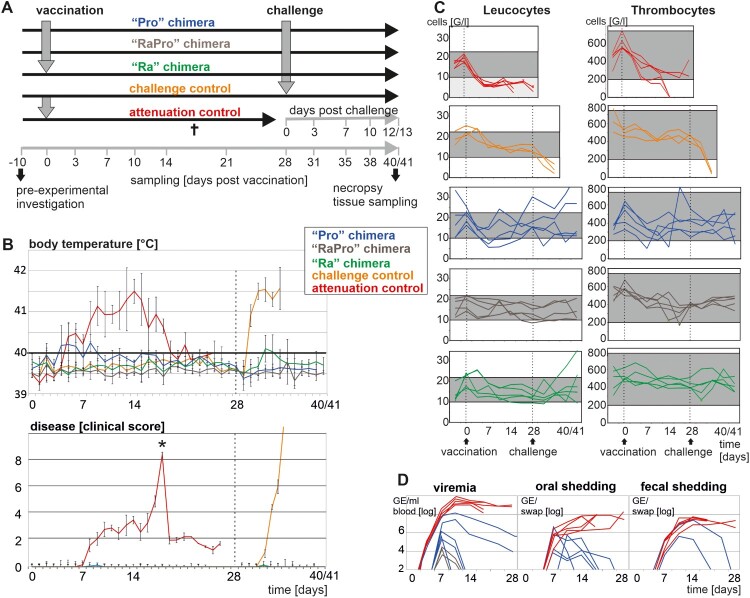
*In vivo* characterization of chimeric vaccine candidates. (A) Experimental design. Groups of five pigs were vaccinated each, only the unvaccinated group (challenge control) consisted of three animals. Time points of sampling are indicated below the time axis. Three severely diseased animals of the attenuation control group were sacrificed 18 dpi (†) and finally sampled. (B) Body temperature (top) and clinical presentation (bottom). Clinical scores were calculated as previously proposed by Mittelholzer [20] with slight modifications as described earlier [16]. (C) Time course of blood cell parameters. Reference ranges for leucocytes (10–22 G/l) and for thrombocytes (201–737 G/l) are highlighted in grey. (D) Genome equivalents (GE) of vaccine candidates in the blood and shedding via saliva and faeces.

Pigs were clinically examined and rectal body temperature was measured daily. Clinical scores were calculated as described earlier [16] based on the scoring previously proposed by Mittelholzer [20]. Oral and fecal swaps were re-suspended in one millilitre of cell culture medium containing antibiotics (Penicillin/Streptomycin). Full blood was taken for serum preparation and anti-coagulated blood samples for determination of white blood cells at 12 time points throughout the experiment (Figure 2(A)). Leucocyte and thrombocyte counts were determined using a haematology analyser (Abacus Junior vet/130464, Guder Medizintechnik, Bad Oeynhausen, Germany). On the day of euthanasia, each animal was subjected to post-mortem examination and organ and serum samples were taken.

### Detection of CSFV genomes, antibodies and quantification of infectivity

For detection of CSFV genomes, RNA was isolated from medium of re-suspended fecal and oral swap samples or EDTA-blood after one freeze–thaw cycle using the QIAamp Viral RNA Mini Kit (Qiagen, Hilden). For RNA isolation from tissue samples a NucleoSpin RNA Kit (Macherey/Nagel) was used as recommended. To determine the CSFV genome loads quantitatively, an accredited CSFV specific real-time PCR was applied using a QuantiTect Probe RT–PCR mastermix (Qiagen, Hilden) with primers and probe previously established [21].

For detection of CSFV E2- and E^rns^- specific antibodies the IDEXX CSFV Ab Test (IDEXX Laboratories, The Netherlands) and the *pigtype* CSFV E^rns^ Ab ELISA kits (Indical Biosciences, Germany) were used according to the manufacturer protocols, respectively. For quantification of neutralizing antibodies (nAbs), three independent titrations (each in duplicates) were performed with the parental CSFV strain Alfort-p2477 (genotype 2.3) and the heterologous challenge virus strain Koslov (genotype 1.1) according to the procedure established at the EU and OIE Reference Laboratory for CSF [19].

## Results

### Generation and in vitro characterization of chimeric viruses

Successful replacement of the original E^rns^ encoding sequence in the cDNA of CSFV Alfort-p2477 was confirmed by PCR and subsequent nucleotide sequencing. Transfection of *in vitro* transcribed RNA into porcine SK6 cells resulted in expression of viral proteins as shown by immunofluorescence testing. The “Pro” chimera completely infected a porcine cell culture monolayer like the parental CSFV strain Alfort-p2477. Further analysis revealed that the “Pro” chimera replicated to titers comparable to Alfort-p2477 (Table 1). The “Ra” chimera showed a significantly reduced replication efficiency and was only able to infect single small foci. Repeated passages of the “Ra” chimera did not significantly increase the number and size of infected foci and the titers remained low at about 10^4.4^ TCID_50_/ml. To achieve a chimeric virus with intermediate properties *in vitro*, the E^rns^ sequence was chimerized itself (Figure 1(A)). The resulting chimera, designated “RaPro”, was generated with the aim to increase the replication of the “Ra” chimera. The Pronghorn pestivirus E^rns^ encoding sequence demonstrated to be advantageous for the replication properties in the used CSFV backbone (Figure 1(B)). After several passages of “RaPro” on different cell lines titers increased to 10^5.3^ TCID_50_/ml (Table 1). Determination of the complete genome sequences of the three chimera revealed no mutations in the genome of the “Pro” chimera, but several changes throughout the genomes of the “Ra” and “RaPro” chimera (Figure 1, **supplementary Table**).

**Table 1.** Characteristics of CSFV strains and vaccine candidates.

Group	CSFV controls		Vaccine candidate		
	Challenge (Koslov)	Attenuation (Alfort-p2477)	“Ra”	“RaPro”	“Pro”
Spread *in vitro*	Continuous	Continuous	Small foci	foci	Continuous
Titer [TCID_50_/ml]	10 ^5.8^	10 ^6.1^	10 ^4.4^	10 ^5.3^	10 ^6.0^
Volume [ml/pig]	2.0	1.0	3.2	1.0	1.0
Dose [TCID_50_/pig]	10 ^6.7^	10 ^4.60^*	10 ^5.05^*	10 ^4.75^*	10 ^4.64^*
Route [vaccination/challenge]	—/oral	i.m./—	i.m./oral	i.m./oral	i.m./oral

*Inoculum titration of vaccine virus stock, diluted to contain 10^5.0^ TCID_50_.

### Attenuation and induction of immunity against classical swine fever

To characterize the properties of the chimeric viruses in the natural host, an animal experiment was performed (Figure 2(A)). Pigs inoculated with the parental virus Alfort-p2477 (attenuation control) showed severe clinical signs of CSF. Three pigs had to be sacrificed at 18 days post infection (dpi) and the other two pigs showed a less severe course of disease until end of the experiment (27 dpi). In contrast, intramuscular (i.m.) application of the “Ra”, “Pro”, and “RaPro” chimera did not result in any clinical signs of CSF (Figure 2(B)). Therefore, all chimera displayed clear attenuation. With the exception of two of the five animals vaccinated with the “Pro” chimera, all other animals vaccinated with the three chimera did not show increased body temperatures.

Although challenge was performed with a very high dose (10 ^6.7^ TCID_50_/pig) of the most virulent CSFV strain available, none of the vaccinated animals showed fever or any other signs of disease after challenge infection (Figure 2(B)). In the group vaccinated with the “Ra” chimera a slight increase of rectal temperature was observed a few days after challenge infection at 28 days post vaccination (dpv). In contrast, all three animals of the unvaccinated challenge control group developed very high fever already on the second day after challenge infection, displayed severe signs of CSF and had to be euthanized at 6 dpi (Figure 2(B)). In conclusion, application of the three vaccine candidates resulted in solid protection against disease after high dose challenge with a genetically heterologous (genotype 1.1) and highly virulent CSFV.

### Blood cell parameters of vaccinated pigs

Blood cell counts were used as an additional parameter to monitor the degree of attenuation of the three CSF vaccine candidates compared to the parental Alfort-p2477 (Figure 2(C)). As expected, unvaccinated pigs showed dramatic decline of leucocytes and thrombocytes within 3–6 dpc resulting in final thrombocyte counts of 40–65 G/l and leucocytes ranging between 2.1 and 6.6 G/l. A similar drop in cells was observed in three animals of the attenuation control group that had to be sacrificed at day 18 after infection with CSFV Alfort-p2477. The two remaining pigs that developed a less severe course of CSF still showed critical leucocyte counts (3.0–5.3 G/l), but thrombocytes were in the physiological range. Among the vaccinated animals, variation of leucocyte and thrombocyte counts was the highest in the “Pro” group. Three animals showed a slight, transient leucopenia at one to four sampling time points. Most of the pigs showed also a slight decline of thrombocytes, but only two animals had cell counts below the physiological range for one single day at 7 or 14 dpv, respectively. In the “RaPro” chimera vaccination group two animals showed only slightly reduced leucocyte counts at several time points. In the “Ra” chimera vaccination group no specific changes in white blood cell parameters became evident. After challenge infection, two animals (“Ra” and “Pro” group) responded with a very high leucocyte number of 35 and 41 G/l, respectively.

### Systemic spread and shedding of vaccine candidates via saliva and feces

To investigate the potential of the vaccine candidates to cause viremia and to be excreted, a real-time RT–PCR was used for viral genome detection and quantification (Figure 2(D)). Viral genomes could be detected in blood of all animals infected with the parental CSFV strain Alfort-p2477 already at 3 dpi. Maximum genome loads of 10^9^–10^10^ genome equivalents (GE)/ml blood were reached at ten days post i.m. application. Interestingly, two animals inoculated with the “Pro” chimera showed a simultaneous increase, however, reaching lower genome loads (10^7^−10^8^ GE/ml). Genome levels decreased from 10 dpv on, but both animals were still genome positive at 28 dpv. Although not showing any clinical signs of CSF, these two pigs were the same animals that showed initial increase of body temperatures. The other three animals of the “Pro” vaccinated group were genome positive in the blood samples only at days 7 and 10 and with lower genome loads ranging between 10^5^ and 10^6^ GE/ml. All five pigs of the “Pro” inoculated group transiently shed viral genomes via saliva between 7 and 21 dpv, but only the two pigs with the long term viremia also shed viral genomes with the feces. In the “RaPro” vaccine study group low amounts of viral genome were detectable in the blood of only three animals. No genome was detected in any of the oral and fecal swap samples. All five animals inoculated with the “Ra” chimera were tested genome negative in blood, saliva and feces, which is consistent with the low viral replication observed in cell culture.

### Post-mortem investigations and presence of viral genomes in tissues

Pathological alterations providing evidence for CSF were absent in all vaccinated pigs. In addition, lymphatic tissues of the head comprising tonsil and *lymphnodus mandibularis*, the parotic gland as well as spleen and kidney tissues were analysed for the presence of vaccine and challenge virus genomes (Table 2). All tissues obtained from pigs of the control groups were highly positive for CSFV genome. In the vaccinated groups, most CSFV genome positive samples and highest genome loads were detected in the “Pro” vaccinated group. Two genome positive spleen samples originated from the two animals that showed a long-term viremia after vaccination with the “Pro” chimera. Direct nucleotide sequencing of a PCR amplicon comprising a 5′NTR fragment revealed that spleen samples contained genomes of the “Pro” vaccine and of the challenge strain, respectively. In the tonsils of the “Pro” vaccine group, only the genome of the vaccine virus was detectable. This might be explained by the lack of sensitivity when performing Sanger sequencing of PCR amplicons that might result in the lack of detection of low amounts of the challenge virus. Nevertheless, at least the majority of viral genomes are obviously originating from the “Pro” chimera. The still high abundance of replicating “Pro” chimera at the time point of challenge might have prevented a subsequent challenge infection by a mechanism known as superinfection exclusion phenomenon. In the group vaccinated with the “Ra” chimera, only the mandibular lymph nodes of all five pigs and the tonsils of four animals were virus genome positive. A representative set of tissues obtained from this group, comprising all PCR positive tonsils and one lymph node sample, was analysed by nucleotide sequencing and revealed to contain only challenge virus genome. In the “RaPro” vaccination group, virus genome was detectable only in two of five pigs. Low genome loads were present in mandibular lymph node samples of both pigs and in one tonsil sample. Sequencing revealed only the presence of challenge virus genome in these tissues (Table 2).

**Table 2.** Detection of CSFV genomes in selected tissues.

Group	Tissue				
	*Ln. mand.*	Tonsil	Spleen	Kidney	Parotis
“Pro”	+	+	+	+	−
	+	+	+	−	−
	+	+	−	−	−
	+	+	−	−	−
	−	+	−	−	−
“RaPro”	+	+	−	−	−
	+	−	−	−	−
	−	−	−	−	−
	−	−	−	−	−
	−	−	−	−	−
“Ra”	+	+	−	−	−
	+	+	−	−	−
	+	+	−	−	−
	+	+	−	−	−
	+	−	−	−	−
Attenuation	+	+	+	+	+
Control	+	+	+	+	+
	+	+	+	+	+
	+	+	+	+	+
	+	+	+	+	+
Challenge	+	+	+	+	+
Control	+	+	+	+	+
	+	+	+	+	+

Note: Representative positive tissues (+) of vaccinated animals were analysed by nucleotide sequencing to discriminate between vaccine strain (blue) and challenge virus (orange).

### Antibody response after application of vaccine candidates and DIVA properties

**Figure 3.**
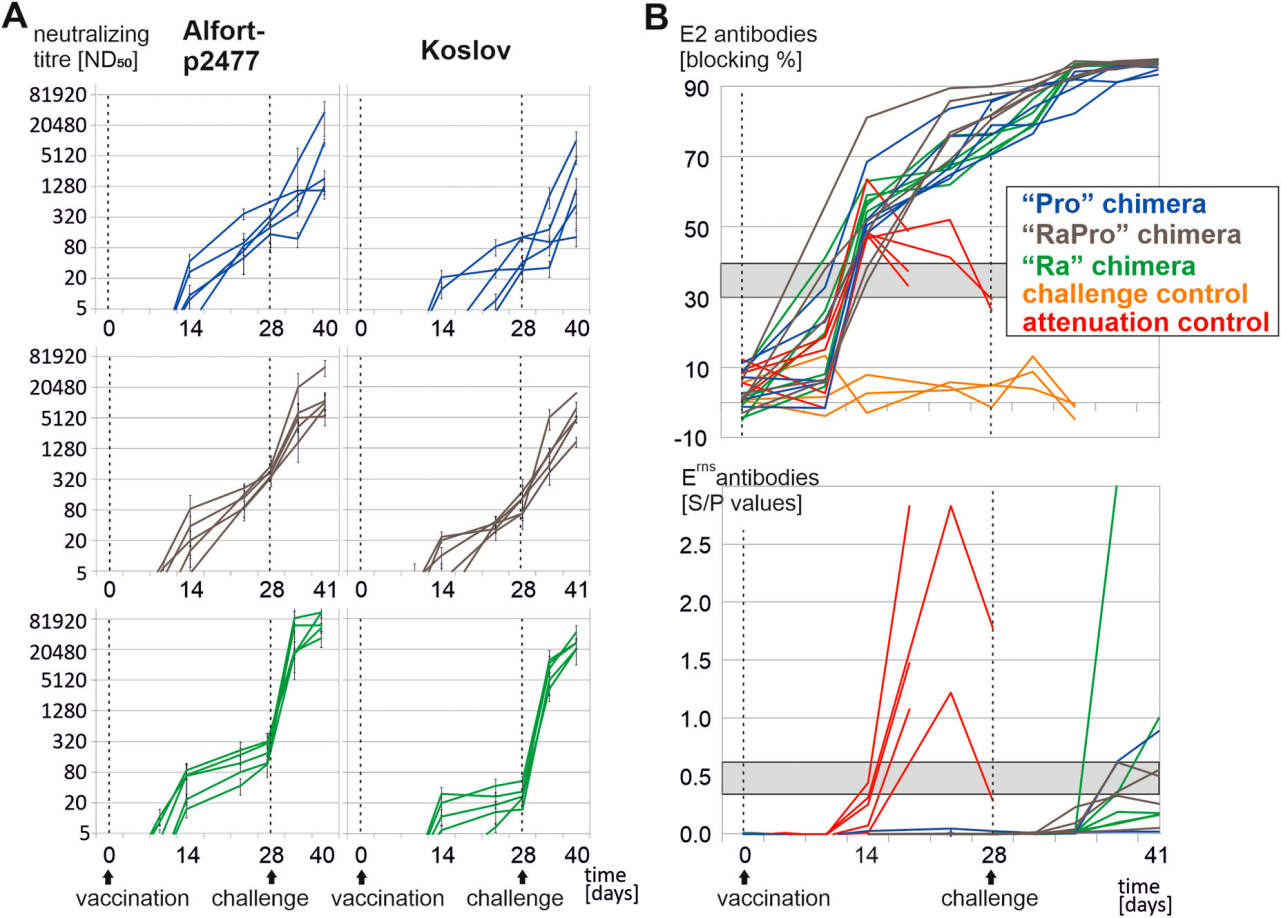
Characterization of the humoral immune response and DIVA properties. (A). Mean neutralizing titers [ND_50_] of three independent titrations and standard deviations. The three rows show the kinetics of neutralizing Ab induced after vaccination with the “Pro”, “RaPro” and “Ra” chimera and subsequent challenge infection. Virus neutralization was performed with the homologous parental CSFV strain Alfort-p2477 (genotype 2.3) and the heterologous highly virulent challenge virus Koslov (genotype 1.1). (B) Seroconversion and DIVA properties of the vaccine candidates. Detection of CSFV specific antibodies was performed by conventional E2 Ab ELISA (upper panel) and DIVA E^rns^ Ab ELISA (lower panel). Shaded boxes indicate doubtful ranges in the respective ELISAs; values above are positive and values below are negative.

To determine the induction of nAbs, serum samples were taken regularly during the experiment for subsequent analysis of virus neutralization against the parental CSFV strain Alfort-p2477 (genotype 2.3) and the genetically heterologous challenge strain Koslov (genotype 1.1) (Figure 3(A)). To be able to investigate the capability to induce nAbs, challenge infection was performed at a relative late time point at 28 dpv. The highest variation with regard to the first time point of detection and titers of nAbs was observed in the animals inoculated with the “Pro” chimera. Neutralizing Abs (ND_50_/ml ≥ 10) were first detectable between 14 and 21 dpv and homologous titers ranged between 147 and 649 ND_50_/ml (28 dpv). In the “Ra” vaccination group, nAbs were detected as early as 10–14 dpv, but titers were slightly lower in average reaching 113–320 ND_50_/ml (homologous virus) and 15–53 ND_50_/ml (heterologous virus). The “RaPro” chimera induced nAb between 10 and 21 dpv. The average titers ranged between 347 and 587 ND_50_/ml (homologous virus) and 67–180 ND_50_/ml (heterologous virus) and thus were highest among the vaccinated groups. After challenge infection, a dramatic increase of titers was observed in all vaccinated pigs. At 12/13 dpc (final sampling) average titers were determined highest in the “Ra” vaccination group (up to 109,227 ND_50_/ml), lowest in the “Pro” vaccination group (up to 35,840 ND_50_/ml), while intermediate neutralization titers were detected in animals vaccinated with the “RaPro” chimera (Figure 3(A)).

Most importantly, the DIVA properties were investigated using an E^rns^ antibody ELISA as accompanying test and a conventional E2 antibody (Ab) ELISA for detection of CSFV specific Ab (Figure 3(B)). All vaccinated pigs showed very high reactivity in the E2 Ab ELISA with more than 70% blocking at time point of challenge. Nevertheless, none of the samples taken from the fifteen vaccinated pigs prior to challenge infection was reacting positively in the E^rns^ Ab based DIVA ELISA, independently on the used vaccine candidate. Five animals developed an E^rns^ specific Ab response already 7–10 dpc with strain Koslov, while for the remaining animals E^rns^ specific antibodies were not detectable until the end of the experiment at 12 or 13 dpc.

## Discussion

Vaccination is a powerful tool in the control and eradication of CSF, if embedded in a suitable overall strategy tailored to the local conditions of pig production. The ideal marker vaccine would combine the early onset of immunity and efficacy of MLVs with the safety and a robust serological negative marker of subunit vaccines.

The presented study was performed to design and characterize MLV candidates with improved marker properties. For this purpose, chimerization of CSFV with only distantly related pestiviral sequences was performed. The replication properties of the three generated chimeric viruses *in vitro* correlated with the degree of attenuation in the host. Nevertheless, all vaccine candidates conferred solid protection against clinical signs of CSF at 28 dpv. In pigs vaccinated with CP7_E2Alf, a full clinical protection against challenge with the same highly virulent CSFV (strain Koslov) was reported to occur already after 1 week and partial protection against a moderately virulent strain already as early as 2 dpv [22,23]. Early onset of protection can be a critical parameter, if vaccination is performed in an emergency situation during an outbreak. The onset of immunity was not in the focus of this initial study and remains to be investigated. Time point of challenge infection was at 28 dpv to be able to investigate induction of humoral immunity and to address possible cross-reactivity of induced Ab responses. So far, available MLVs with marker properties have been constructed by chimerization of CSFV genomes with BVDV sequences (Flc-LOM-BE^rns^) or vice versa (CP7_E2Alf). At least for the latter it has been shown in different studies that cross-reactive Abs are induced that interfere with a robust negative serological marker for DIVA [8,9,24,25]. Comprehensive data about robustness of the DIVA principle Flc-LOM-BE^rns^ vaccine are lacking. Cross-reactivity is likely to occur, although not reported in a study investigating seroconversion in a limited number of animals [11]. The use of CSF marker vaccines in domestic pigs and bait vaccination of wild boar in South Korea just started very recently [12]. It will be of great interest to learn how robust this DIVA concept works under field conditions. Nevertheless, when developing a next generation of marker vaccines focus should be on an improved DIVA concept.

Starting point of the presented study was the hypothesis that genetically distinct pestiviruses might open an avenue to create replicating chimeric viruses with improved marker properties due to significantly reduced risk of inducing cross-reactive Ab. In the past, numerous experimental CSF marker vaccine candidates have been generated and evaluated, all of them based on sequences of closely related pestiviruses [26]. It can be assumed that chimerization of CSFV genomes with E^rns^ encoding sequences of only distantly related pestiviruses probably results in a non-functional genome or a poorly replicating virus. This assumption is in line with the finding of a previous patent study (WO2017114778A1). The inventors claimed that replacement of the 3′ region of an E^rns^ gene from a pestivirus that is genetically closely related to the mutant pestivirus will be required to achieve a well replicating chimera. In the presented study, it was demonstrated that the CSFV genome can tolerate the replacement of the E^rns^ encoding sequence by only distantly related sequences. Replacement of the complete CSFV E^rns^ by the Pronghorn E^rns^ resulted in a very well replicating chimeric virus. In a previous study, chimerization of the genome of BVDV strain NADL with the E^rns^ coding sequence of the Pronghorn pestivirus led to reduced replication compared to the parental strain and chimera containing the E^rns^ of the more closely related giraffe and reindeer pestiviruses, respectively [27]. So far, the NrPV pestivirus E^rns^ sequence is the genetically most distinct sequence used to design pestivirus MLVs with marker properties. Low similarity and limited compatibility with the CSFV backbone may explain that the chimera replicated poorly in cell culture. However, it was possible to improve *in vitro* replication by replacing the 3′ portion of the NrPV pestivirus E^rns^ encoding sequence by the homologous sequence of Pronghorn pestivirus. The observation that *in vitro* replication of the “RaPro” chimera increased after passaging on cell culture is probably due to acquisition of adaptive mutations. Occurrence of mutations during cell culture passaging is a helpful mechanism to improve replication of artificially constructed genomes of RNA viruses. In this line, multiple mutations have been previously described in the CP7_E2Alf chimera, including one important amino acid change in the E^rns^ reported to be responsible for efficient replication in porcine cells [7]. The amino acid changes at position T4I and S130R of the “RaPro” E^rns^ are different to the mutations observed in CP7_E2Alf and another mutation known to be important for acquired heparan sulfate binding during cell culture adaptation [28]. Of note, the polar, uncharged serine (S) at position 130 is frequently found in BVDV strains, but the acquired basic arginine residue (R) is highly conserved only in CSFV sequences. The acquired arginine residue may contribute to the improved viral replication in porcine cells.

Although all three chimeric viruses were attenuated, the introduction of heterologous sequences into the CSFV Alfort-p2477 backbone had different impact on the replication properties not only *in vitro,* but also *in vivo*. Virus genomes of other MLVs can consistently be detected in tonsils up to day 42 and in blood samples within the first 14 dpv by real-time RT–PCR [29]. Thus, a certain degree of replication is a common feature of MLVs. Nevertheless, the “Pro” chimera would require additional attenuation to obtain a safe vaccine. In contrast, the “Ra” vaccine candidate showed very strong attenuation, as it was not possible to detect any viral genomes in the blood, saliva, feces or any of the analysed tissues. Based on all parameters investigated, the “RaPro” chimera showed an intermediate phenotype and best balance between replication and attenuation. The absence of challenge virus genome in tonsils and mandibular lymph nodes of most pigs vaccinated with the “RaPro” chimera already 12 days after oral infection demonstrates the power of the induced degree of protection. In a study comparing the efficacy of different CSFV marker vaccine candidates, some pigs showed viral genome positive tonsils still at 49 days after oro-nasal challenge with CSFV Koslov, but were not infecting contact animals [30].

Although high amounts of CSFV Ab were induced, sera of all vaccinated pigs – independently on the applied vaccine candidate – revealed to be non-reactive in the CSFV E^rns^ Ab based DIVA assay. Further studies, including a higher number of vaccinated animals and repeated vaccination, would be necessary to determine differences in the robustness of the three introduced serological negative markers. Due to higher antigenic distance to CSFV E^rns^, the NrPV and “RaPro” E^rns^ antigens can be expected to be superior in serological DIVA application compared to the Pronghorn pestivirus E^rns^.

Taken together, combination of CSFV genomes and genetically distinct E^rns^ encoding sequences allows designing chimeric attenuated viruses that can be propagated in amounts suitable for vaccine production. Chimeric MLVs containing E^rns^ encoding sequences of distantly related pestiviruses are a very promising strategy for establishing an improved serological negative marker in CSF marker vaccines.

## Supplementary Material

suppl-Table_R1_clean.docx
