# Supplementary material for: Genetically distinct pestiviruses pave the way to improved classical swine fever marker vaccine candidates based on the chimeric pestivirus concept
Source: Emerg Microbes Infect. 2020 Oct 3;9(1):2180–9. doi: 10.1080/22221751.2020.1826893 (PMC7580611; doi:10.1080/22221751.2020.1826893)
Supplement: suppl-Table_R1_clean.docx [file TEMI_A_1826893_SM5365.docx]

**Supplementary Table: Genetic characterization of chimeric CSFV vaccine candidates “Ra” and “RaPro” after continued cell culture propagation**

| **Genomic region** | **Position* of mutations in chimeric genomes** | |
| --- | --- | --- |
|  | **“Ra” chimera** | **“RaPro” chimera** |
| N^pro^ | - | 758T>C |
| E^rns^ ***** | 383G>A (R128K)  667T>G (S222A) | 11C>T (T4I), 383A>G, 388A>C (S130R) |
| E1 | 2097C>T (H575Y) | - |
| NS2-3 | 3978T>C, 6257G>A | 4001C>T |
| NS4B | 7855A>G (N2494S) | 7540C>G (A2389G) |
| NS5A | - | 9197G>T (E2941D) |
| NS5B | 10456C>A (T3361N) | - |
| 3’NTR | - | 12175_12176insTATT |

Numbering of the CSFV sequences is according to the reference sequence of CSFV Alfort-p447 (GenBank LT593760) and provides information about position in the genome and in the deduced polyprotein sequence (given in brackets). *Numbering of mutations in the E^rns^ region refers to the positions in the E^rns^ encoding sequence and the deduced amino acid in E^rns^ protein. Positions in the E^rns^ sequence of the “Ra” chimera refer to the E^rns^ sequence of the Norway rat pestivirus (NrPV). Numbering in the sequence of the “RaPro” chimera is different due to differences in length of the NrPV and Pronghorn pestivirus E^rns^ sequences. Thus, numbering of “RaPro” E^rns^ is referring to the position in the chimeric E^rns^ encoding sequence. For non-synonymous mutations, changed amino acids are given in brackets.
